# Computer-Aided Screening for Potential Coronavirus 3-Chymotrypsin-like Protease (3CLpro) Inhibitory Peptides from Putative Hemp Seed Trypsinized Peptidome

**DOI:** 10.3390/molecules28010050

**Published:** 2022-12-21

**Authors:** Kansate Prasertsuk, Kasidit Prongfa, Piyapach Suttiwanich, Nathaphat Harnkit, Mattanun Sangkhawasi, Pongsakorn Promta, Pramote Chumnanpuen

**Affiliations:** 1 Pibulwitthayalai School, 777 Naraimaharach, Talaychoopsorn, Lopburi District, Lopburi 15000, Thailand; 2 Medicinal Plant Research Institute, Department of Medical Sciences, Ministry of Public Health, Nonthaburi 11000, Thailand; 3 Program in Biotechnology, Faculty of Science, Chulalongkorn University, Bangkok 10330, Thailand; 4 Omics Center for Agriculture, Bioresources, Food and Health, Kasetsart University (OmiKU), Bangkok 10900, Thailand; 5 Department of Zoology, Faculty of Science, Kasetsart University, Bangkok 10900, Thailand

**Keywords:** antiviral, peptide, hemp, SARS-CoV-2 main protease, molecular docking

## Abstract

To control the COVID-19 pandemic, antivirals that specifically target the severe acute respiratory syndrome coronavirus 2 (SARS-CoV-2) are urgently required. The 3-chymotrypsin-like protease (3CLpro) is a promising drug target since it functions as a catalytic dyad in hydrolyzing polyprotein during the viral life cycle. Bioactive peptides, especially food-derived peptides, have a variety of functional activities, including antiviral activity, and also have a potential therapeutic effect against COVID-19. In this study, the hemp seed trypsinized peptidome was subjected to computer-aided screening against the 3CLpro of SARS-CoV-2. Using predictive trypsinized products of the five major proteins in hemp seed (i.e., edestin 1, edestin 2, edestin 3, albumin, and vicilin), the putative hydrolyzed peptidome was established and used as the input dataset. To select the *Cannabis sativa* antiviral peptides (csAVPs), a predictive bioinformatic analysis was performed by three webserver screening programs: iAMPpred, AVPpred, and Meta-iAVP. The amino acid composition profile comparison was performed by COPid to screen for the non-toxic and non-allergenic candidates, ToxinPred and AllerTOP and AllergenFP, respectively. GalaxyPepDock and HPEPDOCK were employed to perform the molecular docking of all selected csAVPs to the 3CLpro of SARS-CoV-2. Only the top docking-scored candidate (csAVP4) was further analyzed by molecular dynamics simulation for 150 nanoseconds. Molecular docking and molecular dynamics revealed the potential ability and stability of csAVP4 to inhibit the 3CLpro catalytic domain with hydrogen bond formation in domain 2 with short bonding distances. In addition, these top ten candidate bioactive peptides contained hydrophilic amino acid residues and exhibited a positive net charge. We hope that our results may guide the future development of alternative therapeutics against COVID-19.

## 1. Introduction

The severe acute respiratory syndrome coronavirus 2 (SARS-CoV-2) that first emerged in late 2019 (known as COVID-19), can cause severe pneumonia in humans. This coronavirus has a high spread rate and has had tremendous impacts on multiple facets of society, human health, and economics. Although there have been several efforts to rapidly develop vaccines or repurpose small-molecule inhibitors against SARS-CoV-2 [1,2], there are no generally proven effective therapies for this particular disease [3]. Recently, peptides and peptide-based inhibitors have been considered as compelling alternatives to small molecules since they can be chemically and biotechnologically synthesized. Because of the rapid procedure for the design and development of therapeutic peptides, the newly discovered candidates can compete with the speed of virus strain evolution. Moreover, the functional peptides have a unique mode of action and show specific activity on the molecular and cellular targets compared to other small-molecule drugs [4,5,6]. 

There have been several proposed drug targets to inhibit SARS-CoV-2 focusing on their entering and amplification in host cells. Some examples include structural proteins such as the glycosylated spike (S) protein (which mediates recognition by the host cell receptor, called angiotensin-converting enzyme 2 (ACE-2)) and the group of non-structural proteins, i.e., RNA-dependent RNA polymerase (RdRp), the CoV main protease (Mpro) or 3-chymotrypsin-like protease (3CLpro), and papain-like protease (PLpro) [7]. The replication process of SARS-CoV-2 is mediated by a complex of two polyproteins that are translated from the viral RNA [8]. During replication in the host cell, the viral proteases 3CLpro and PLpro must process their polyproteins to form the functional viral replication complex [9]. Hence, both 3CLpro and PLpro are proposed as the major logical targets to inhibit the replication of SARS-CoV-2, and the 3CLpro inhibitor could represent a valid potential therapeutic strategy for COVID-19 treatment [8,10,11]. It has been suggested that 3CLpro is one of the significant factors, and if we can find the main protease inhibitory peptide, the rate of the coronavirus pandemic and the number of virus strains will be lessened [8,12,13,14]. In general, the coronavirus 3CLpro structure contains three domains: domain I (residues 8–101), domain II (residues 102–184), and domain III (residues 201–303) [8]. This enzyme has a Cys–His catalytic dyad with the substrate-binding site located between domains I and II. Hence, the desired 3CLpro inhibitor should potentially interact with the amino acid residues of the highly conserved substrate-recognition pocket of this enzyme active site (residues 8–184) [8,15].

Hemp seed is emerging as an alternative plant protein source because of its rich protein content and reasonable amino acid profile, containing 20–25% protein, depending on environmental factors [16,17]. The specific globulin protein, edestin, is the most abundant protein in hemp seed, which together with albumin, the second-most predominant, account for 60–80% of the protein; vicilin is found in very low amounts compared to other seed-accumulated proteins [16,18]. The pleiotropic health-promoting effects of hemp seed protein hydrolysate are promising, and numerous investigations (both computational and experimental approaches) have sought to better understand the beneficial effects on health of these putative and derivative peptides [18]. There have also been many in silico and in vitro studies and reports on the biological activities of hemp seed peptides, such as peptides with lipid-lowering, cholesterol-lowering [19], antioxidative [20,21,22,23,24], antihypertensive [21,25], anti-inflammatory and immunomodulatory [26], hypoglycemic [27], and allergenic activities [28]. The first indications of the potential of cannabis on COVID-19 treatment was the study by Orio et al., revealing the inhibitory effect of four hemp seed peptides that could bind to the ACE-2 (the host cell receptor) to protect it from the viral S protein attachment [25,29]. Even though there were some functional peptides candidates for COVID-19 from several plant species to inhibit the coronavirus’ necessary proteins, the inhibitory peptides from hemp seed against the 3CLpro of SARS-CoV-2 have never been reported [30,31,32]. Currently, bioinformatic pipelines and in silico screening are becoming alternative preliminary approaches for the discovery and development of drugs to counter COVID-19 (via 3CLpro inhibition) and have the benefits of cutting costs and speeding up the process before in vitro and in vivo validations [33,34,35].

Therefore, the present study screened for potential inhibitors of the major functional protein of SARS-CoV-2, 3CLpro, from hemp seed putative trypsinized peptidome using a bioinformatic approach. Molecular docking and molecular dynamics were employed to validate the interaction of the 3CLpro–csAVP complex.

## 2. Results and Discussion

### 2.1. Hempseed Putative Antiviral Peptides Screening Using Computational Method

**Figure 1:**
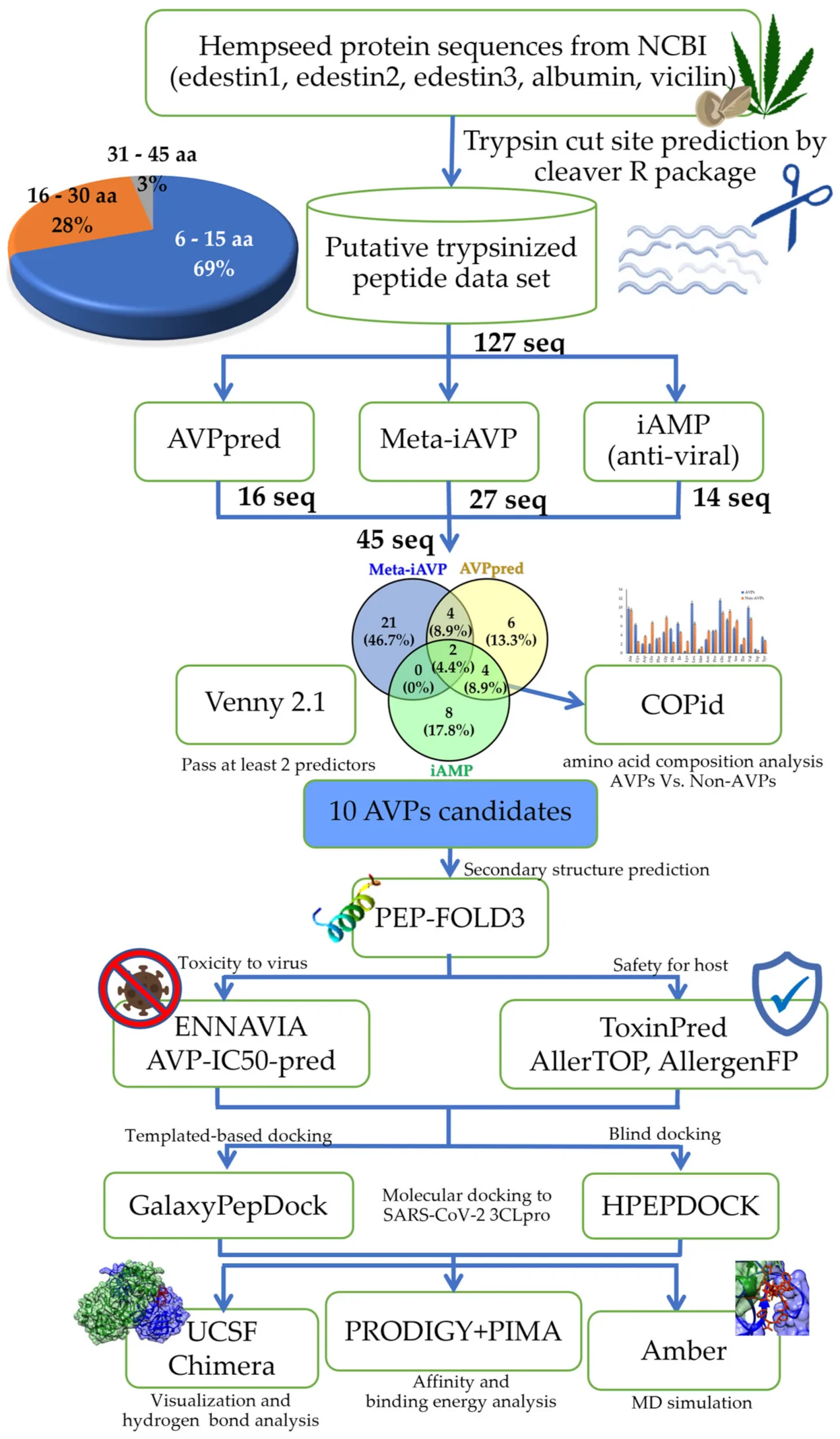
The workflow of the computer-aided virtual screening for the *Cannabis sativa* antiviral peptides (csAVPs) candidates and the in silico validation of the selected coronavirus 3CL protease inhibitory peptides using molecular docking and molecular dynamic simulation.

**Figure 2:**
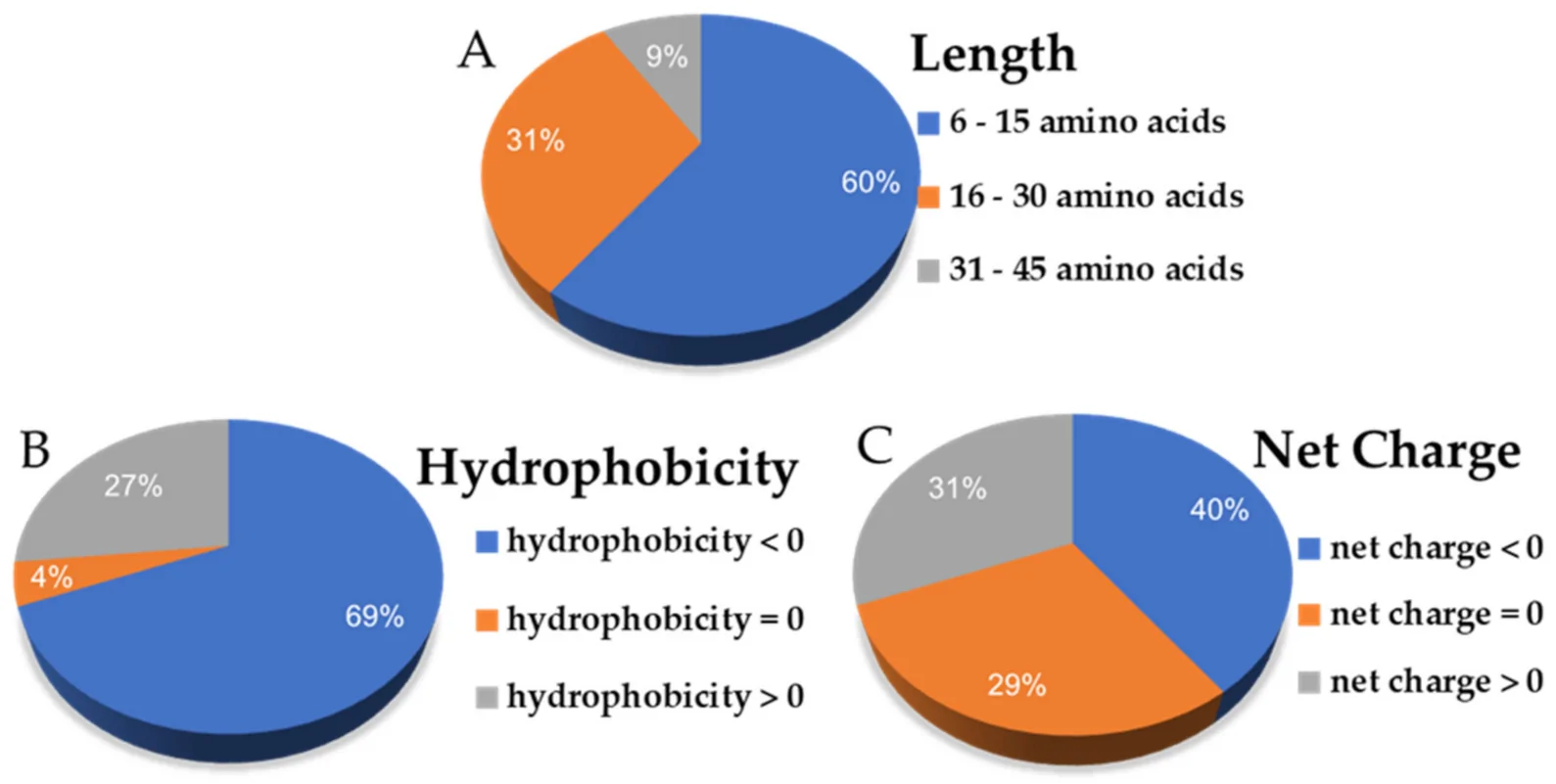
The distribution ratio in each class of general peptides features such as peptide length (**A**), hydrophobicity (**B**), and net charge (**C**) from 45 putative csAVPs.

The input dataset of hemp seed (*Cannabis sativa*) peptide sequences as the putative trypsinized results of five main proteins (edestin1–3, albumin, and vicilin) is listed in the Supplementary file (Table S1). For our selection constraints, *Cannabis sativa* antiviral peptides (csAVPs) were selected based on the cut-off criterion that the predicted score had to be larger than half of the total values. For example, the support vector machine (SVM) probability must be over 50 in AVPpred (either in amino acid composition-based or physicochemical property-based models) and the probability must be greater than 0.5 in Meta-iAVP and iAMPpred (focusing on the antiviral classification). Based on these constraints, 16, 27, and 14 AVPs were predicted by AVPpred, Meta-iAVP, and iAMPpred, respectively. From the dataset of 127 input hemp seed peptide sequences, there were 45 sequences passing at least 1 AVP predictor, and only 10 of these peptides could pass at least 2 prediction servers (Figure 1 and Table 1). The major csAVP candidate source was the trypsinized products from vicilin protein (5 selected csAVPs), while the rest were from edestin2, 3 and albumin. Notably, there was no selected csAVP from the edestin1 protein source at all. The majority (more than 60%) of these 45 putative csAVPs were categorized as short (6-15 amino acids) and hydrophilic peptides based on their overall characteristics and abundance (Figure 2). Notably, the peptide net charge of AVPs were quite equally distributed into negatively charged, positively charged, and non-charged groups, as shown in Figure 2. 

According to the information in the length distribution of 45 csAVP candidates, there were 27 (60%) and 14 (31%) peptides classified as short AVPs (6–15 residues) and medium AVPs (16–30 residues), respectively. Compared to other reported AVPs, the lengths of AVPs are quite variable, ranging from short to medium in length, containing 8–40 amino acid residues, and consisting of positively charged side chain amino acids [36,37,38,39,40,41]. In general, the recommended length for AVPs is between 10 and 40 amino acid residues, which could be used as a guideline for the AVPs length optimization to improve antiviral peptide properties [42,43]. According to the calculated physicochemical properties (Table 2), only five csAVPs (csAVP5, 7–10) are cationic peptides based on the net charge value. However, all csAVPs are considered as amphipathic peptides containing positively charged side chain amino acids, either arginine (R) and/or lysine (K). The amphipathic property of AVP has been suggested to be significantly influenced by the charge distribution profile around the polar–nonpolar interface, which can inhibit virus-induced cell fusion [44]. Even though the molecular function and mechanism of amphipathic antiviral peptides on coronavirus 3CL protease (3CLpro) has not been illustrated before, several amphipathic peptides were investigated through in silico and in vitro experiments as the potential 3CLpro inhibitors [31,32,45,46,47]. 

**Figure 3:**
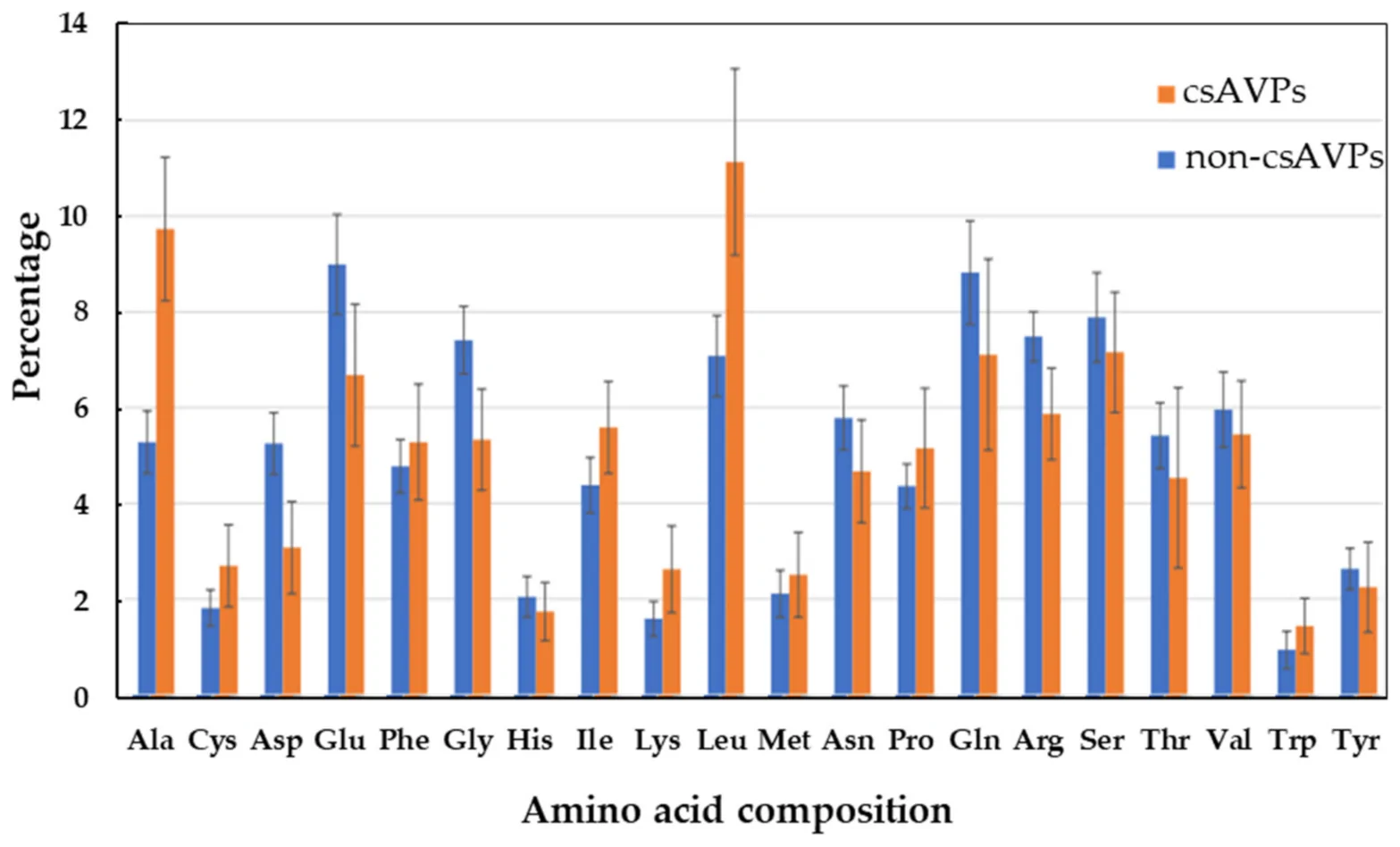
The comparative analysis of amino acid composition represents the preferences between the predicted csAVPs and non-csAVPs from hemp seed putative peptidome.

The compositional analysis of csAVP amino acid sequences is shown in Figure 3. According to the analyzed result, the hydrophobic side chain amino acids (i.e., Ala, Leu, Ile, Lys, Cys, and Trp) were found in higher percentages compared to the non-csAVPs. Notably, the positively charged side chain amino acids (Lys and Arg) are still found in moderate abundance. This is reasonable due to the fact that these functional antimicrobial peptides are usually amphipathic, that is, composed of hydrophobic side chains and cationic residues at the same time [36,48,49]. In particular, the basic side chain residue (Lys) plays a really important role in the electrostatic properties of antiviral peptides and is commonly found as a preferential residue in therapeutic peptides [36]. Moreover, Lys provides the cationic property of the AVPs, enhancing the antiviral activity by facilitating the cell surface–peptide interaction and leading to the insertion into microorganisms, either through the anionic cell walls or phospholipid membranes [36,48,50,51]. Notably, in our case, Lys was not found to be a preferential amino acid of csAVPs, and the molecular mechanism of Lys toward the inhibition of viral enzymes has never been clearly revealed [36,52]. These putative csAVPs might not be as specifically involved in membrane–peptide interaction as other AVPs. Noticeably, the aliphatic and medium-sized hydrophobic side chain amino acids (Ala, Cys, Leu, and Ile) were also found to have higher percentage compositions in csAVPs. These non-polar residues have been reported to play a significant role in the amphipathic characteristics of antimicrobial peptides [36,53].

### 2.2. Predictive Antiviral Scores, IC_50_, and Physicochemical Properties of the Selected csAVPs

All ten selected csAVPs, classified as probable antivirals by at least two prediction programs with the cut-off as mentioned in the previous section, are listed in Table 1. All details about each candidate, including secondary structures, peptide sequences, and protein origin, are provided together with the predictive scores from four machine learning-based predictive programs. Focusing on the predicted secondary structures of each peptide, one of the most important peptide sequence features for predicting AVPs, peptide folding prediction of all selected csAVPs, was performed by the PEP-FOLD3 webserver. Our chosen peptides can be divided into three structural groups: helix-consisted loop (csAVP1, 4, 5, 6, and 8), random coiled (csAVP2, 3, 7, and 10), and β-sheet-consisted loop (csAVP9). This finding is consistent with the previous research reports that random coils and α-helices are the two major classes of AVP secondary structures, as opposed to the β-sheet structure [31,36,54]. 

Briefly, the red numbers indicate the significant predictive scores that pass our cut-off criteria, as mentioned before (over 50 in SVM scores for AVPpred and over 0.5 probability for Meta-iAVP and iAMPpred). Compared to the prediction result from the AMPfun (http://fdblab.csie.ncu.edu.tw/AMPfun/index.html (accessed on 10 February 2022)) web server in the previous work by our group [31], the iAMPpred tended to be more strict as an AVP screening tool. Since the first two models of AVPpred program (antiviral peptide motif-based and sequence alignment-based models) could only provide classification results (either AVPs or non-AVPs), we only consider the predictive SVM scores from Model 3 (M3) and Model 4 (M4) in the csAVP candidate selection procedure. The algorithms of these two models were based on amino acid compositions (M3), and physiochemical properties (M4), respectively. For the ENNAVIA program, both modes of the neural network prediction models (antiviral and anticoronavirus) were performed. The first two models (ENNAVIA-A and B) were used for antiviral property classification, while the other two models (ENNAVIA-C and D) were specifically used for anti-coronavirus property prediction [55]. 

To access information about the antiviral activity of our selected csAVPs, the AVP-IC_50_Pred server was used to predict the AVP half maximal inhibitory concentration (IC_50_), which is the major inhibition profile. Since SARS-CoV-2 was not listed on the “virus specific” prediction platform, the RSV/INFV/HSV prediction model was selected as the closest virus choice. The other two hybrid models were also selected to predict the inhibitory doses for viruses in general, and the average predicted IC_50_ (µM) was shown in the last column of Table 2. The particular calculation value relied on a regression-based algorithm, and various peptide features (i.e., amino acid composition, binary profile, physicochemical properties, and solvent accessibility) were considered to predict the IC_50_ value of the AVP sequences [56]. From the virus-specific SVM model prediction, all csAVPs were found to have quite comparable predicted IC_50_ (45–48 µM), whereas the virus-specific RF model gave a wider range of predicted IC_50_ (14.62–366.97 µM). The higher predicted IC_50_ of csAVP1 and csAVP5 above 46 µM in the virus-specific SVM model indicated lower antiviral efficacy compared to other peptide candidates. However, csAVP5 and 4 were found to have the highest efficacy among putative antivirals based on the average IC_50_ across three prediction models, with only 24.50 and 29.90 µM, respectively.

In the previous study on AVP screening for the human angiotensin-converting enzyme 2 (ACE-2) inhibitor, they divided the antiviral efficacy into four levels based on the estimated IC_50_ values: highly effective (<1 μM), effective (1–10 μM), moderately effective (11–100 μM), and least effective (>100 μM) [30]. Our selected csAVPs could be classified as moderately effective based on the predicted virus-specific SVM model and the average IC_50_ values. However, all csAVPs could be classified as highly effective (<1 µM) if only the Hybrid Model II IBk and K Star models were considered, as could csAVP1 and csAVP6 based on the Hybrid Model I K Star model. Based on the virus-specific SVM model, the predicted antiviral efficacy of our selected csAVPs is comparable to that of the selected fruit bromelain-derived peptide for human angiotensin-converting enzyme 2 (ACE-2) inhibition (40.67 µM), whereas the virus-specific RF values of our csAVPs (14.62–366.97 µM) are higher than that of the bromelain-derived peptide (6.85 µM) [30]. It should be noted that these predicted IC_50_ values are only predictions; in vitro testing is still required to validate the real efficacy of these candidates.

Furthermore, Table 3 has provided information about the physicochemical property calculated scores of all selected csAVP candidates (csAVP1 to csAVP10) obtained from the ToxinPred webserver. The smallest peptide candidate was AVP10, with a molecular weight of 835.99 g/mole, while csAVP4 was the largest candidate, with a molecular weight of 4131.18 g/mole. Consistent with the previous study, all selected putative csAVPs were characterized as amphipathic functional peptides with high steric hindrance and sidebulk scores (over 0.5) [31]. 

### 2.3. In silico Toxicity and Allergenicity Analysis of the Selected csAVPs

To obtain the high specificity and low side effects of the bioactive peptides, both toxicity and allergenicity are usually considered when designing effective and safe therapeutics. Aside from laboratory-based approaches, in silico screening of non-toxic peptides has recently been found to be the most efficient way to further verify the specificity and selectivity of functional peptides [57]. The estimation of probable toxic peptides was performed by the computational predictive tool, ToxinPred, to estimate the possible side effects of csAVPs on the host cell. In particular, ToxinPred analysis was based on eight different algorithms: SVM (Swiss-Prot)-based, SVM (Swiss-Prot) + Motif-based, SVM (TrEMBL)-based, SVM (TrEMBL) + Motif-based, Monopeptide (Swiss-Prot), Monopeptide (TrEMBL), Di-peptide (Swiss-Prot), and Dipeptide (TrEMBL). The predictive SVM scores of each model can be observed in Table 4. The negative values indicate the non-toxic classified results, while the positive values indicate the possible toxicity of the analyzed peptides (highlighted in red). Only one selected peptide (csAVP3) was classified as a probable toxic peptide in some predictive models (model A and B). The ToxinPred prediction values were negative for the remaining candidates, indicating a very low toxigenic potential for host cells. 

Compared to the previous work of our group [31], only the toxicity side effects of the bioactive peptide candidates were considered in the potential AVP screening. To determine another possible side effect, bioinformatic tools for allergenicity prediction were employed in this study. Allergenic peptide identification is a crucial parameter for the development of vaccines and therapeutic peptides. Both AllerTOP v.2.0 and AllergenFP v.1.0 prediction tools could estimate the allergenicity factor of the polypeptides using amino acid sequence-based predictive algorithms. The prediction by the AllerTOP v2.0 server was based on an amino acid sequence and a physicochemical property-based machine learning model developed for allergenic peptide/protein classification [58]. In contrast, AllergenFP can distinguish the allergenic from the non-allergenic peptide by an alignment-free, descriptor-based fingerprint method [59]. The accuracy of the AllerTOP and AllergenFP programs were stated as 85.3 and 88%, respectively [58,59]. According to the allergenic prediction results in Table 4, only four csAVP candidates (csAVP4, 5, 6, and 8) were classified as non-allergenic peptides, while the rest were identified as probable allergenic peptides either by AllerTOP or AllergenFP servers (highlighted in red). It should be noted that the estimation value of the peptide property does not guarantee the activity of the candidate peptide in its real use. The efficacy of functional peptides can be influenced by several factors in real biological systems (i.e., ions, digestive enzymes, pH, temperature, and solvent tonicity). Therefore, laboratory experiments are still required for further validation of all selected peptide candidates to ensure their non-toxic and non-allergenic effects. Moreover, the redesign and optimization of some residues can be considered as a guideline to improve the efficiency, safety, and specificity of csAVP candidates in the future. 

### 2.4. Molecular Docking of csAVPs with SARS-CoV-2 3CL Protease

The process of bioinformatics prediction and screening for the coronavirus 3CL protease (3CLpro) inhibitory peptides is quite challenging due to the limitations of the specific in silico screening tools available. Since there was no “ideal perfect docking program” that could give the highest accuracy and best performance in all cases, we tried to employ the best docking program in both docking approaches. According to the comparative study of 14 docking programs on protein–peptide complexes by Weng et al. [60], GalaxyPepDock (as a template-based docking approach) performs the best compared to other template-based docking programs and significantly better than any template-free docking programs. HPEPDOCK, on the other hand, performs the best and is more computationally efficient for global docking compared to other programs. To estimate and rank the binding energy and affinity of all csAVP candidates on 3CLpro, both programs were selected to perform the molecular docking in this study.

**Figure 4:**
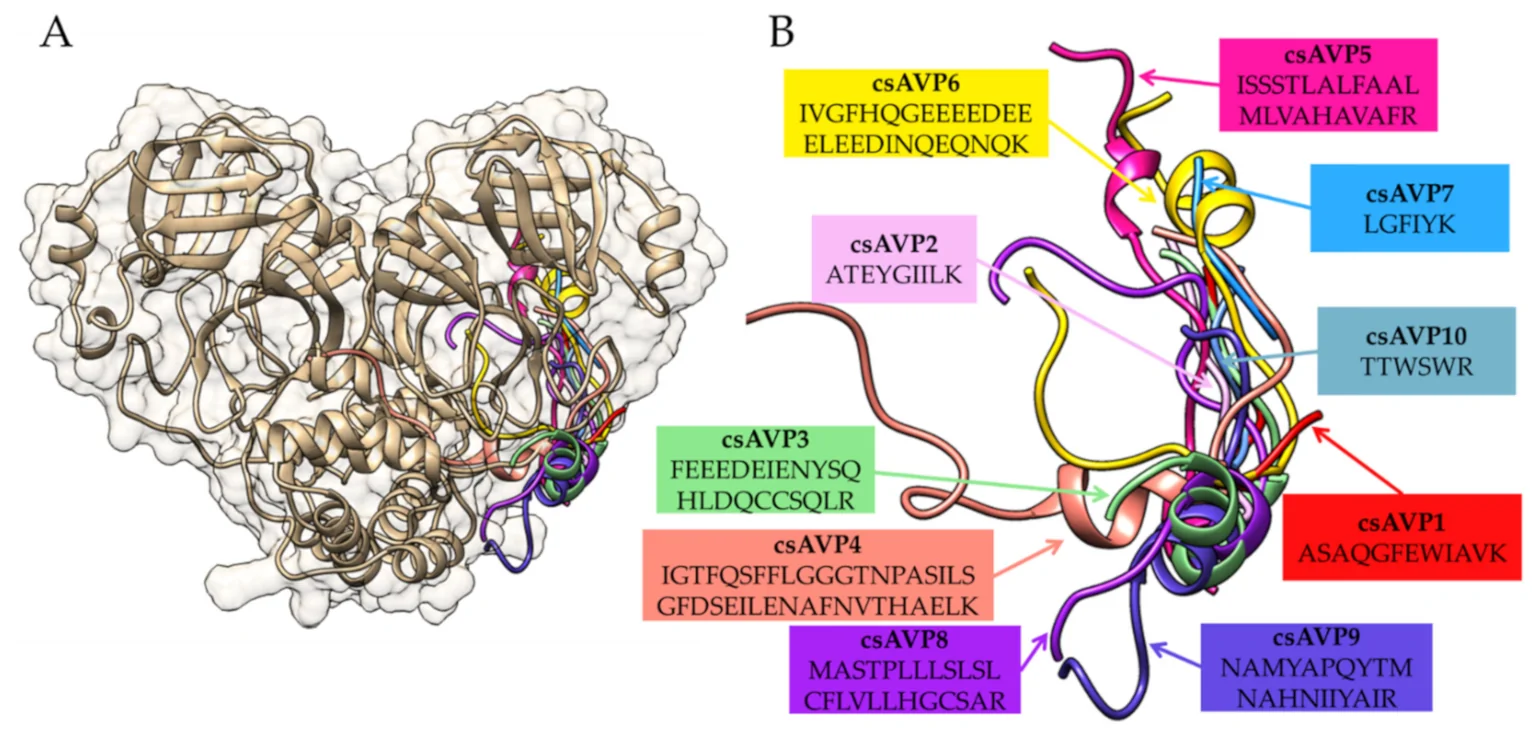
Comparison of molecular docking position of all selected csAVPs (csAVP1 to csAVP10) on the crystal structure of coronavirus 3CL protease in the apo state (PDB ID: 7C2Q) shown with (**A**) and without (**B**) the enzyme structure. Structure of the coronavirus 3CL protease is shaded in gold and the csAVPs are labeled with different colors.

**Figure 5:**
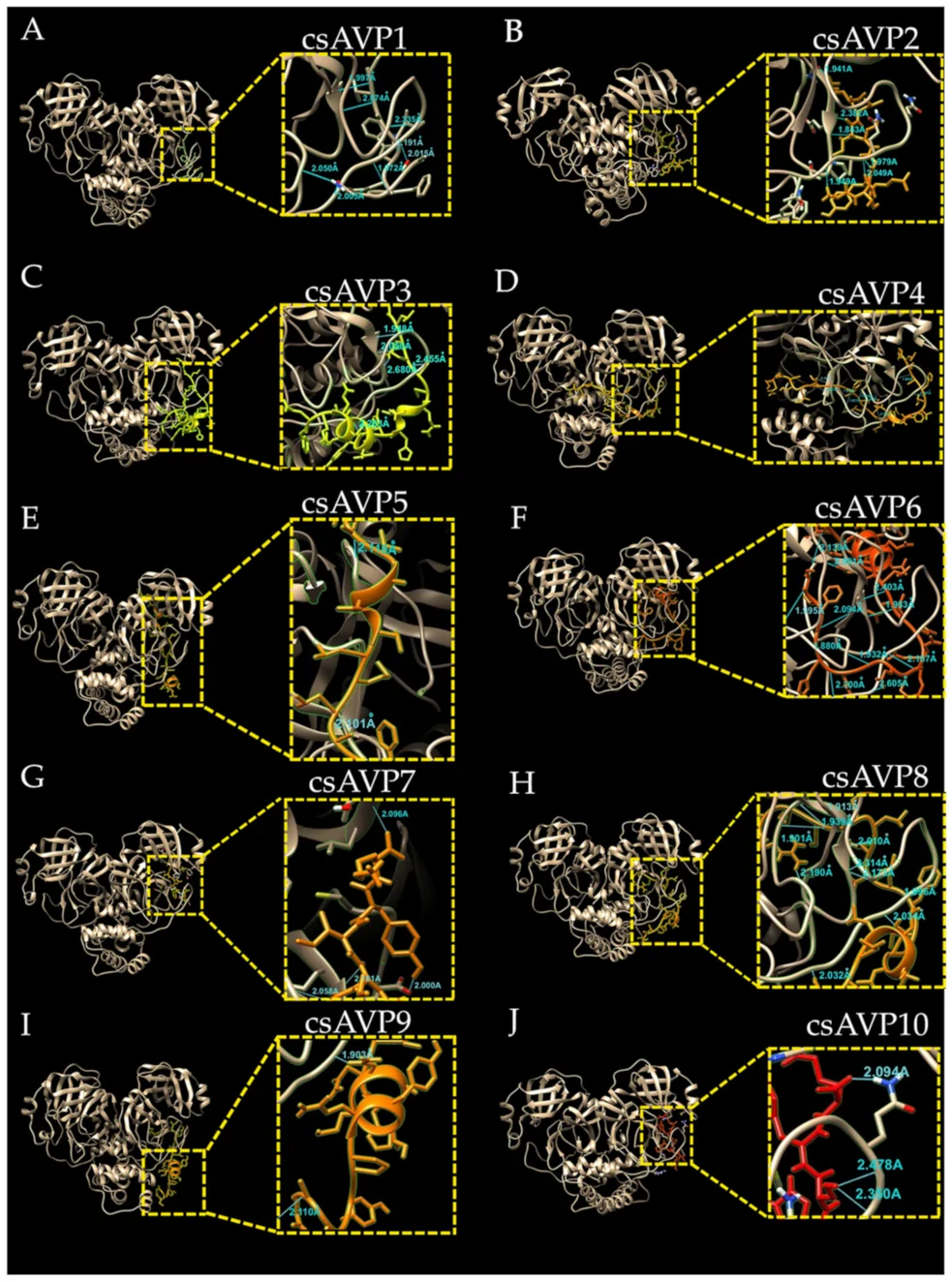
Molecular docking of the ten selected csAVPs (csAVP1-10) to the crystal structure of Coronavirus 3CL protease in the apo state (PDB ID: 7C2Q) (**A**–**J**). Structure of the coronavirus 3CL protease is shaded in gold, and the peptide chains are labeled with the csAVP number above. The blue lines indicate the hydrogen bonds with the value of bonding distance.

The molecular docking simulation of these ten selected csAVPs to the apo state of the coronavirus 3CLpro crystal structure suggested the ability of csAVP1 to csAVP10 to bind near the substrate binding groove of the SARS-CoV-2 3CLpro structure (Figure 4). To compare the docking areas of different AVPs on the SARS-CoV-2 3CLpro structure, all csAVP candidates shared common binding positions on the active site groove (Figure 4). The interaction between the side chains of these selected csAVPs and the side chains near the active site of the SARS-CoV-2 3CLpro with hydrogen bond formation was observed (Figure 5). All hydrogen bonds observed between the candidate peptides (csAVP1 to csAVP10) and the binding pocket of coronavirus 3CL protease in the protein–peptide docking simulation are listed in Table 5. In particular, csAVP6 and -4 (the largest peptide candidates) could form ten and nine hydrogen bonds (mainly interacted with Arg123 and Glu134 residues) near the 3CLpro binding pocket, respectively. Compared to the previous research reports, the docking positions of all selected csAVPs closer to the catalytic site area of the SARS-CoV-2 3CLpro structure were very similar to the docking positions of the known 3CLpro inhibitory marine polyketides [61], antiviral drugs [62], and rice bran AVP candidates [31]. 

Focusing on the bonding energy types, hydrogen bonds with donor-acceptor distances of 2.2–2.5 Å are considered as “strong, mostly covalent”, while those of 2.5–3.2 Å and 3.2–4.0 Å are considered as “moderate, mostly electrostatic” and “weak, electrostatic", respectively [63]. According to the interaction between the enzyme (3CLpro) and our selected inhibitors (csAVPs), the most optimized hydrogen bond acceptor–donor pair is usually found in the distance between 2.7 and 3.3 Å [64], and most hydrogen bonds between peptide molecules and protein backbones are starting to be considered as strong binding from a 3 Å bonding distance [65]. Based on our molecular docking simulation experiments, the distance in the hydrogen bond between selected csAVPs and SARS-CoV-2 3CLpro structures was found to range from 1.8 to 2.7 Å. In particular, the selected peptides tend to interact with Glu134, Gln177, Thr178, and Gln180 residues of SARS-CoV-2 3CLpro by hydrogen bonding. These hydrogen bonds were observed in relatively short bonding distances (1.8–2.5 Å), indicating the strong, mostly covalent interactions, and representing the high binding affinity. 

Focusing on the calculated scores of binding affinity and binding energy obtained from PROGIDY and PIMA servers (Table 6), csAVP6 showed the strongest binding to the active site area of the SARS-CoV-2 3CLpro with a molecular docking score of −572.17 kJ/mol and a binding affinity (ΔG) of −15.6 kcal/mol. These docking scores were calculated based on the template-based docking results from the GalaxyPepDock server [66]. Interestingly, the largest peptide candidate (csAVP4, as the top two in docking score at −447.27 kJ/mol) showed the highest binding affinity of −18.2 kcal/mol with the best dissociation constant of 4.30 × 10^−14^ among all csAVPs. These significant high docking scores and binding affinity were promisingly better compared to the docking scores on coronavirus 3CLpro of several antiviral drugs, i.e., noscapine, chloroquine, ribavirin, and favipiravir with the scores of −292.42, −269.71, −214.17, and −153.91 kJ/mol, respectively [62]. Focusing on the docking scores obtained from another famous blind assessment of a protein−protein docking server (HPEPDOCK as a template-free docking tool) [67], csAVP4 was ranked as one of the top-four, while csAVP6 showed the least docking ability among all csAVPs. At this point, csAVP4 seemed to be the best candidate for further analysis based on the overall docking parameters and the non-toxic and non-allergenic characteristics. The 3CLpro-csAVP4 interface interactions involving affinity and binding energy distribution was obtained by high van der Waals energy −420.90 kcal/mol (hydrophobic interactions) and hydrogen bond energy −41.90 kcal/mol, which are considered as the most significant calculation to assess the binding stability. Based on the molecular docking results of this study, it was proven that the selected csAVPs (especially csAVP4 with the best overall docking results) could be strong potential candidates for SARS-CoV-2 3CLpro inhibitors in controlling COVID-19 disease. 

### 2.5. Molecular Dynamics Simulation of 3CLpro-csAVP4 Complex 

Since molecular docking and molecular dynamics methods can provide such valuable insights regarding the physicochemical properties of bioactive molecules, they are commonly used as a virtual screening strategy. These simulations can provide information about potential drug candidates’ interactions and reactivity with protein targets. As discussed in the previous section, the binding affinity docking scores, non-toxicity, and non-allergenicity were taken into consideration, and csAVP4 was selected as the most promising candidate to be a coronavirus 3CLpro inhibitor. Thus, the molecular dynamics simulation of csAVP4 binding to the 3Clpro backbone was performed for 150 nanoseconds to examine the conformational stability and fluctuation analysis of the complex. The stability of the csAVP4 peptide and 3Clpro complex was estimated by RMSD, Rg, and RMSF trajectory analysis.

**Figure 6:**
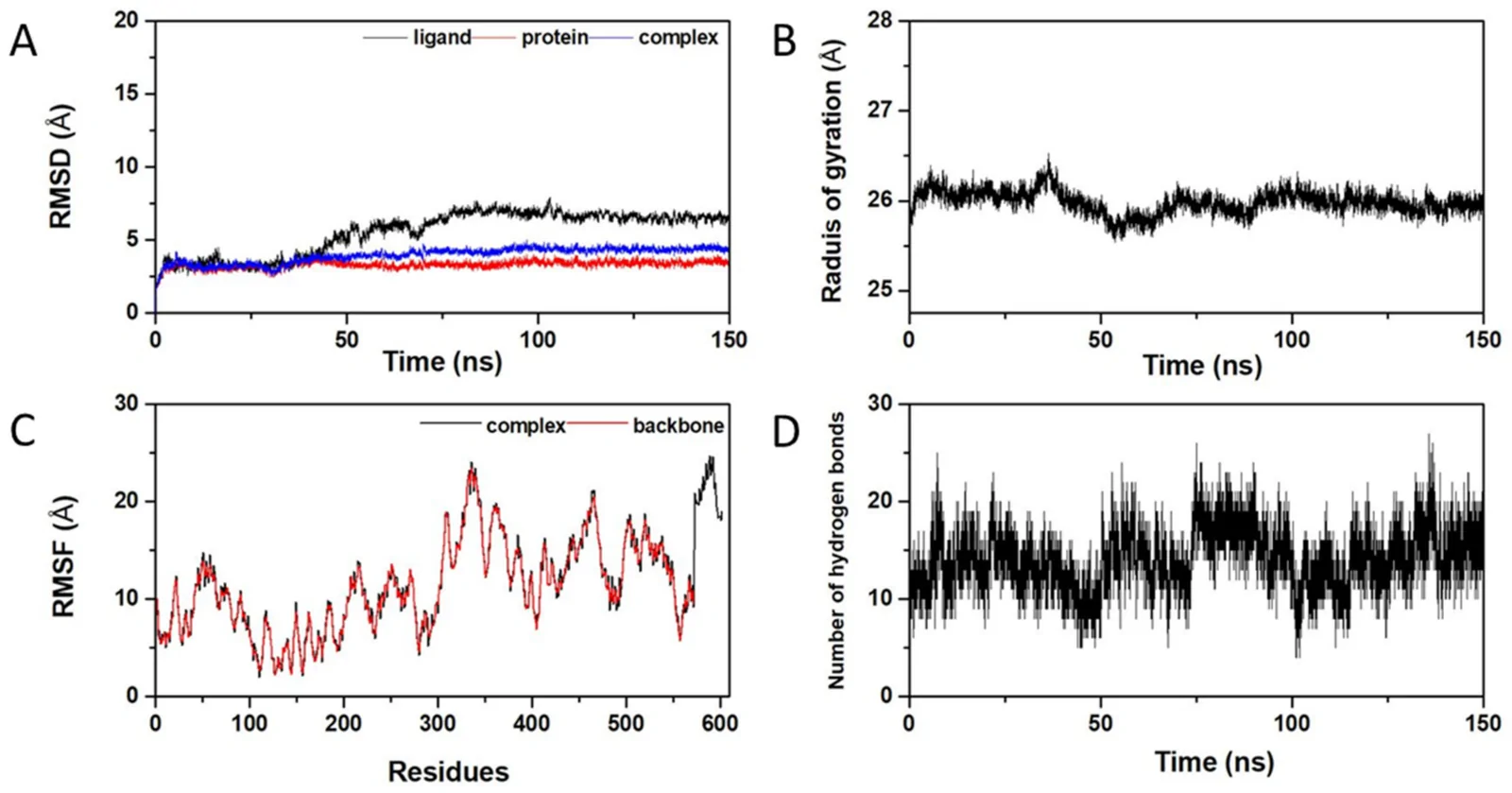
Molecular dynamic simulation of coronavirus 3CLpro–csAVP4 complex: (**A**) RMSD profiles of the csAVP4 ligand (black) 3CLpro protein backbone (red) and RMSD profiles of 3CLpro–csAVP4 complex (blue); radius of gyrus plot of the 3CLpro–csAVP4 complex (**B**); RMSF profile of the 3CLpro–csAVP4 complex (**C**); and hydrogen bonds plot for the 3CLpro–csAVP4 complex (**D**).

The analysis of the root-mean-square deviation (RMSD) profile is necessary to define the compactness of protein after the ligand-induced fit into the protein complex [68,69]. In particular, the simulation of the RMSD profile is based on the atomic coordinates of backbone atoms from the protein and ligand trajectories. The low fluctuation pattern of the RMSD profile represents the higher stability of the interested protein–peptide complex [70]. The RMSD values of the csAVP4 ligand, 3CLpro protein backbone, and their complexes were remaining stable in the range of 3-6 Å (Figure 6). As shown in Figure 6A, the 3CLpro complex with csAVP4 was quite rigid, with less than 4 Å RMSD (blue line), and they had a similar trend along the 150 ns simulation time as the apo form of the protein backbone (red line) and the csAVP4 ligand (black line) for the last period of dynamics. The result indicated that the enzyme–inhibitor complex remained stable after a certain period of time. To define the structural activity of the enzyme–inhibitor complex, the radius of gyration (Rg) of the involved trajectories was also simulated (Figure 6B). The Rg value fluctuated according to the folding state of the 3CLpro–csAVP4 complex. Low fluctuations were observed in the range of 25.5-26.5 Å, indicating the stability of the 3CLpro protein backbone while binding with csAVP4.

After that, the RMSF profiles of the 3CLpro–csAVP4 complex were also generated to determine the conformational stability of the protein–peptide complex (Figure 6C). The low fluctuation of coordinates in the range of 5-25 Å indicates the high stability of the protein–peptide complexes. Lastly, the hydrogen bond involvements were also analyzed to estimate the dynamic equilibration of the 3CLpro–csAVP4 complex. The hydrogen bonding profile with a high number of hydrogen bonds during the simulation period indicated the stable binding of csAVP4 with the target 3CLpro enzyme (Figure 6D). In conclusion, these molecular dynamics profiles demonstrated the prolonged and robust binding of csAVP4 to the target coronavirus 3CLpro and the involvement of potential binding energies with the correlation of molecular dynamics profiling and the stability of the 3CLpro−csAVP4 complex.

### 2.6. Similarity of the csAVP4 against the Known Anti-Coronavirus Peptides Database

To ensure that the sequence of the selected bioactive peptide has not been reported as an anti-coronavirus peptides (ACovPs) before, the csAVP4 sequence was used as a query peptide to search against the anti-coronavirus peptides database (ACovPepDB) [71]. Using the ACovPBLAST option, the matching results of csAVP4 to the eight ACovPs stored in ACovPepDB are listed in Table S2. Even though the %identity of the short focusing sequence was high (26–100%), the percentage of matching residues (%Matching) calculated from the number of residues matched on csAVPs was low (only 10–30%). The matching residues are shown in red, indicating the very short overlap between the query peptide and the known ACovPs. The results indicated that our selected AVP is a potential novel ACovP that has never been reported before. Notably, additional comparative structural analysis and optimization could be used to improve antiviral activity against coronaviruses, specifically via the inhibition of the 3CLpro enzyme.

## 3. Materials and Methods

According to the bioinformatic pipeline in Figure 1, a computer-aided virtual screening workflow with in silico validation by molecular docking and molecular dynamic simulations has been developed and proposed. The workflow begins with predictive digested peptidomes of the five most abundant proteins in hemp seed (*Cannabis sativa*): edestin 1, edestin 2, edestin 3, vicilin, and albumin. Then, the putative trypsinized peptidome was established and used as the input data sets of 127 peptides until the selection of the antiviral peptides with the most proper predicted scores of unique *Cannabis sativa* antiviral peptides (csAVPs) without probable toxicity and allergenicity side effects to the host cells was performed. 

### 3.1. Preparation of the Hemp Seed Putative Hydrolyzed Peptidome

The input dataset of 127 hemp seed peptide sequences was obtained from predicted cut sites of the five major proteins in hemp seed (*Cannabis sativa*: Uniprot taxonomic ID = 3483); edestin 1, edestin 2, edestin 3, vicilin, and albumin (from the National Center for Biotechnology Information: NCBI with specific accession numbers; A0A090DLH8, A0A090CXP8, A0A219D2X4, A0A219D1T7, and A0A219D1L6, respectively). The in silico trypsin digestion was performed by the R package “cleaver” (version: 1.34.1; [72]). The peptide length distribution in this putative peptidome could be classified into 3 groups, i.e., short (6–15 amino acids), medium (16–30 amino acids), and long (31–45 amino acids). In particular, 88 sequences (69%), 35 sequences (28%), and 4 sequences (3%) were classified as short, medium, and long peptides, respectively (Figure 1).

### 3.2. The Computer-Aided Prediction and Screening of Antiviral Peptides

The input dataset from the previous step was arranged in FASTA format and used for the antiviral peptide prediction. To obtain the antiviral prediction scores, 3 machine learning-based prediction programs, i.e., iAMPpred (http://cabgrid.res.in:8080/amppred/ index.html, accessed on 16 January 2022), AVPpred (http://crdd.osdd.net/servers/avppred, accessed on 17 January 2022), and Meta-iAVP (http://codes.bio/meta-iavp/ accessed on 17 January 2022), were employed. To generate a Venn diagram to visualize the unique, dual, and multiple overlapping csAVP candidates among the different bioinformatic predictive programs, Venny 2.1.0. (https://bioinfogp.cnb.csic.es/tools/venny accessed on 20 January 2022) was used. To obtain the probability score of anti-coronavirus activity, all significant AVP candidates were analyzed with the neural network peptide antiviral and anti-coronavirus activity predictor ENNAVIA (https://research.timmons.eu/ennavia, accessed on 20 January 2022) [55]. The average scores from 4 predictive models of ENNAVIA (2 antiviral and 2 anti-coronavirus predictive models) together with the other 3 AVP predictors were considered to select the csAVP candidates. To distinguish between the preferential amino acid types of AVPs and non-AVPs, the amino acid composition (AAC) analysis of the pair group comparison in COPid webserver (http://www.imtech.res.in/raghava/copid/, accessed on 20 January 2022) was employed [73]. Lastly, PEP-FOLD3.0 (https://bioserv.rpbs.univ-paris-diderot.fr/services/PEP-FOLD3, accessed on 22 January 2022) was used to simulate the feasible molecular structure of the candidate antiviral peptides. 

### 3.3. Predictions of Allergenicity, Toxicity, and Physicochemical Properties

The theoretical allergenicity of the peptide was predicted using the Allergen FP v.1.0 (https://ddg-pharmfac.net/AllergenFP/, accessed on 23 January 2022) and AllerTOP v.2.0 (https://www.ddg-pharmfac.net/AllerTOP/, accessed on 23 January 2022) webservers to evaluate whether the selected csAVPs are probable allergens [58,59]. ToxinPred (https://webs.iiitd.edu.in/raghava/toxinpred/protein.php, accessed on 23 January 2022) was used to predict whether the peptides are cytotoxic to the host cells or not. All 4 SVM-based methods with a default threshold value of 0.0 were chosen for predicting the toxicity, and values greater than 0.0 were considered as toxic. All calculated scores of physicochemical properties of selected csAVP candidates were also obtained by the ToxinPred (https://webs.iiitd.edu.in/raghava/toxinpred/protein.php, accessed on 23 January 2022) webserver in the batch submission option, and all possible physicochemical characteristics were selected.

### 3.4. IC_50_ Prediction 

The half-maximal inhibitory concentration (IC_50_) of the peptide’s antiviral activity was predicted using the AVP-IC_50_Pred server (http://crdd.osdd.net/servers/ic50avp/, accessed on 10 February 2022) [36] by selecting RSV/INFV/HSV for virus-specific prediction mode. The AVP-IC_50_Pred server’s Hybrid Models I and II were also used as prediction models for antiviral activity determination with all default setting parameters.

### 3.5. Molecular Docking Simulation of Protein–Peptide 

The three-dimensional structures of all selected csAVP candidates were predicted using the PEP-FOLD 3.5 program (https://bioserv.rpbs.univ-paris-diderot.fr/services/PEP-FOLD3, accessed on 22 January 2022) [74]. The crystal structure of the coronavirus 3CL protease (3CLpro) in the apo state with PDB ID code 7C2Q [75] was accessed from the Protein Data Bank (PDB) (http://www.rcsb.org, accessed on 10 January 2022). 

The amino acid sequences of all selected csAVPs (csAVP1 to csAVP10) were docked to the coronavirus 3CLpro enzyme using both template-based and template-free docking webservers. The template-based molecular docking simulation was performed by GalaxyPepDock (http://galaxy.seoklab.org/pepdock, accessed on 25 January 2022) [75], while the HPEPDOCK (http://huanglab.phys.hust.edu.cn/hpepdock/, accessed on 26 January 2022) was used to estimate the template-free molecular docking scores. The docking results of the best model and hydrogen bond finding were visualized by UCSF Chimera 1.16 program [76]. After that, PIMA webserver [77], available at http://caps.ncbs.res.in/pima (accessed on 1 February 2022), and PRODIGY server [78] (https://wenmr.science.uu.nl/prodigy, accessed on 1 February 2022) were employed to investigate the protein–peptide interface interactions involved in the affinity and binding energy.

### 3.6. Molecular Dynamics Simulations Study

The Amber ff14DB force field and Amber16 software package [79] were used to perform MD simulations of the 3CLpro–csAVP4 complex. The TIP3P water model was used to solvate the system at a distance 10 Å from the protein, and sodium ions were added to neutralize the simulated systems. The initial conformations were heated to 300 K with a canonical ensemble (NVT) for 100 ps before being equilibrated for another 1200 ps. Then, until 300 ns of the production run, all-atom MD simulations were performed under the isothermal-isobaric ensemble (NPT) at 1 atm and 300 K with a simulation time step of 2 fs. The Berendsen barostat [80] with a pressure-relaxation time of 1 ps and the Langevin thermostat [81] with a collision frequency of 2 ps^−1^ were used to maintain pressure and temperature during MD simulation, respectively. The SHAKE algorithm [80] was used to constrain all chemical bonds involving hydrogen atoms, while the particle mesh Ewald’s (PME) summation method [82] was used in the treatment of the long-range electrostatic interactions. The cut-off for non-bonded interactions was set at 10 Å. The CPPTRAJ module of AMBER16 was used to calculate structural analyses in terms of root-mean-square displacement (RMSD), root-mean-square deviation, root-mean-square fluctuation (RMSF), radius of gyration (Rg), and hydrogen bond profile investigated with during the 150 ns.

### 3.7. Similarity Searching of the csAVP4 against the Anti-Coronavirus Peptides Database

The ACovPBlast tool of the peptide database of anti-coronavirus peptides, or ACovPepDB (http://i.uestc.edu.cn/ACovPepDB/index.html, accessed on 30 November 2022), was employed to search for similar peptides stored in the database. The amino acid sequence of csAVP4 was used as the query peptide. The default setting parameters had an expected value of 10 with a maximum result of 300, and the optimized parameters for short peptides (<15 residues) were selected for the blast search.

## 4. Conclusions

In this study, the selected csAVPs from our proposed bioinformatic computer-aided screening workflow were quite varied in length (6–39 amino acid residues) and physicochemical properties. These amphipathic csAVP candidates contain hydrophilic amino acid residues with cationic residues. The molecular docking performances could demonstrate that all csAVP candidates had strong hydrogen bonding with the coronavirus 3CLpro binding groove at Glu134, Gln177, Thr178, and Gln180 residues in domain 2 with strong bonding energy. csAVP4 was selected as the best candidate for coronavirus 3CLpro inhibition among all selected csAVPs based on its predicted efficacy (the second-lowest average predicted IC_50_ among the selected csAVPs), high affinity and binding energy, and low possible side effects to the host cell. Even though the conformational stability of the 3CLpro–csAVP4 complex was confirmed by its molecular dynamics profile, further in vitro and in vivo studies are required to authenticate the anti-COVID-19 potential of these csAVP candidates. 

## Figures and Tables

**Table 1:** Predicted secondary structures, peptide sequences, and antiviral peptide prediction scores from 4 different bioinformatics tools.

Peptide ID	Secondary Structure	Sequences (from Protein)	Length	AVPpred		Meta-iAVP	iAMP pred	ENNAVIA			
				M3 *	M4 *			A *	B *	C *	D *
csAVP1	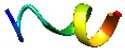	ASAQGFEWIAVK (edestin2)	12	53.13	26.77	0.946	0.527	0.000	0.000	0.000	0.000
csAVP2	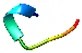	ATEYGIILK (vicilin)	9	43.36	70.76	0.038	0.718	0.001	0.333	0.000	0.325
csAVP3	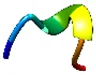	FEEEDEIENYSQHLDQCCSQLR (albumin)	22	53.34	45.49	0.362	0.558	0.128	0.133	0.103	0.154
csAVP4	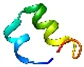	IGTFQSFFLGGGTNPASILSGFDSEILENAFNVTHAELK (vicilin)	39	35.33	64.14	0.542	0.331	0.202	0.000	1.000	0.000
csAVP5	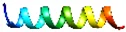	ISSSTLALFAALMLVAHAVAFR (albumin)	22	53.46	70.85	1.000	0.211	0.884	0.000	0.768	0.100
csAVP6	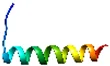	IVGFHQGEEEEDEEELEEDINQEQNQK (vicilin)	27	46.69	64.43	0.282	0.625	0.011	0.000	0.005	0.000
csAVP7	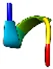	LGFIYK (vicilin)	6	54.22	71.75	1.000	0.859	0.864	1.000	0.199	0.596
csAVP8	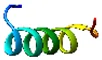	MASTPLLLSLSLCFLVLLHGCSAR (edestin3)	24	49.95	64.74	0.996	0.029	0.888	0.000	0.999	0.000
csAVP9	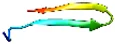	NAMYAPQYTMNAHNIIYAIR (edestin3)	20	37.99	49.6	0.696	0.461	0.422	0.369	0.908	0.502
csAVP10	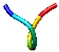	TTWSWR (vicilin)	6	42.03	49.7	0.008	0.655	0.316	1.000	0.000	0.995

Note: For AVPpred program; M3 * = compositions SVM based models (Model 3), M4 * = physiochemical properties SVM-based models (Model 4). For ENNAVIA program, the neural network models were based on antiviral vs. non-antiviral datasets (A *), antiviral vs. random datasets (B *), anti-coronavirus vs. non-antiviral datasets (C *), and anti-coronavirus vs. random datasets (D *). The red numbers indicate the significant predictive scores that pass our cut-off criteria.

**Table 2:** Predictive AVP half maximal inhibitory concentration (IC_50_) by SVM, RF, IBk, and K Star-based models from the AVP-IC_50_Pred webserver.

Peptide ID	Virus Specific		Hybrid Model I *				Hybrid Model II *				Average IC_50_ (µM)
	SVM	RF	SVM	RF	IBk	K Star	SVM	RF	IBk	K Star	
csAVP1	46.17	30.19	99.44	38.00	125.46	0.22	99.26	38.54	0.01	0.01	47.73
csAVP2	45.82	75.32	78.69	108.68	152.62	11.41	78.60	109.07	0.01	0.01	66.02
csAVP3	45.90	111.35	32.97	41.74	58.84	17.01	33.05	42.34	0.01	0.01	38.32
csAVP4	45.92	104.83	40.75	19.24	19.85	8.01	40.74	19.62	0.01	0.01	29.90
csAVP5	48.00	14.62	49.27	19.61	33.46	11.01	49.19	19.84	0.01	0.01	24.50
csAVP6	45.92	247.86	39.43	73.22	35.16	0.01	39.45	71.64	0.01	0.01	55.27
csAVP7	45.79	126.57	46.57	49.41	19.85	313.01	46.56	49.17	0.01	0.01	69.70
csAVP8	45.91	171.33	38.71	13.64	11.80	1.01	38.74	13.84	0.01	0.01	33.50
csAVP9	45.75	366.97	45.99	56.50	36.75	14.01	45.91	56.50	0.01	0.01	66.84
csAVP10	45.92	185.64	45.75	17.61	62.98	9.01	45.73	17.63	0.01	0.01	43.03

Note: Hybrid Model I * = Composition(mono-di) + Physico + Secondary structure + Surface accessibility; Hybrid Model II * = Binary(N8/C8) + Physico + Secondary structure + Surface accessibility.

**Table 3:** Physicochemical properties scores of ten selected csAVPs (csAVP1 to csAVP10) calculated by ToxinPred server.

Peptide ID	Hydrophobicity	Steric Hindrance	Sidebulk	Hydropathicity	Amphipathicity	Hydrophilicity	Net Hydrogen	Charge	pI	Mol wt
csAVP1	0.04	0.62	0.62	0.33	0.52	−0.35	0.58	0.00	6.35	1306.65
csAVP2	0.06	0.64	0.64	0.53	0.55	−0.29	0.56	0.00	6.35	1007.33
csAVP3	−0.34	0.63	0.63	−1.33	0.64	0.58	1.05	−5.50	4.00	2716.18
csAVP4	0.03	0.61	0.61	0.21	0.26	−0.33	0.56	−2.50	4.40	4131.18
csAVP5	0.17	0.56	0.56	1.62	0.18	−0.83	0.41	1.50	10.11	2290.08
csAVP6	−0.36	0.66	0.66	−1.89	0.85	1.12	1.00	−10.50	3.77	3245.71
csAVP7	0.14	0.57	0.57	0.79	0.52	−0.77	0.43	1.00	8.94	868.19
csAVP8	0.12	0.54	0.54	1.43	0.16	−0.81	0.42	1.50	8.40	2546.51
csAVP9	−0.06	0.62	0.62	−0.17	0.26	−0.70	0.85	1.50	8.84	2356.01
csAVP10	−0.27	0.55	0.55	−1.42	0.41	−0.72	1.50	1.00	10.11	835.99

**Table 4:** Predictive probability in toxicity and allergenicity from ToxinPred, AllerTOP, and AllergenFP webservers.

Peptide ID	Toxicity Predictions		Allergenicity Predictions	
	SVM Scores	Toxicity	AllerTOP	AllergenFP
csAVP 1	−0.75	Non-toxin	Probable allergen	Probable non-allergen
csAVP 2	−0.65	Non-toxin	Probable allergen	Probable non-allergen
csAVP 3	0.24	Toxin	Probable allergen	Probable non-allergen
csAVP 4	−1.34	Non-toxin	Probable non-allergen	Probable non-allergen
csAVP 5	−1.18	Non-toxin	Probable non-allergen	Probable non-allergen
csAVP 6	−0.66	Non-toxin	Probable non-allergen	Probable non-allergen
csAVP 7	−1.11	Non-toxin	Probable non-allergen	Probable allergen
csAVP 8	−1.25	Non-toxin	Probable non-allergen	Probable non-allergen
csAVP 9	−0.68	Non-toxin	Probable allergen	Probable non-allergen
csAVP 10	−0.91	Non-toxin	Probable non-allergen	Probable allergen

Note: The red numbers indicate the significant predictive results for probable toxicity or allergenicity of the selected AVPs.

**Table 5:** List of hydrogen bonds observed from molecular docking of ten peptides (csAVP1 to csAVP10) to the crystal structure of coronavirus 3CL protease in the apo state (PDB ID: 7C2Q).

Peptide	Peptide Residues	3CLpro Residue	Distance (Å)	Peptide	Peptide Residues	3Clpro Residue	Distance (Å)
csAVP1	VAL11	GLU134	1.997	csAVP6	GLU15	GLU134	2.403
	VAL11	GLU134	2.074		GLU6	GLU134	1.983
	ALA10	THR178	2.355		PHE4	HIS160	2.094
	TRP8	GLN180	2.191		GLU10	ALA181	2.187
	GLY5	ALA181	1.972		PHE4	ARG123	1.880
	GLN4	ASN125	2.050		HIS5	THR186	2.300
	GLN4	GLY183	2.005		GLY7	ASN125	1.932
	SER2	ALA179	2.015		GLN26	GLY131	2.193
csAVP2	LYS9	ASN111	1.941		GLU17	SER132	2.001
	ILE7	GLU134	2.382		GLU8	THR184	2.605
	ILE7	GLU134	1.843	csAVP7	LYS6	THR24	2.096
	GLY5	GLN180	1.979		TYR5	GLU134	2.000
	TYR4	GLN180	2.049		PHE3	GLU134	2.161
	ALA1	ALA182	1.949		GLY2	GLN177	2.058
csAVP3	GLN20	GLU134	1.948	csAVP8	GLY20	SER132	1.913
	GLN20	GLU134	2.050		GLY20	GLY131	1.939
	SER19	THR178	2.455		LEU17	GLU134	2.010
	TYR10	THR184	2.263		LEU17	GLU134	2.314
csAVP4	GLU26	GLU134	1.905		VAL16	GLU134	2.173
	THR23	GLN180	2.187		PHE14	GLN180	1.996
	SER13	THR127	1.878		ARG24	GLY158	2.190
	PHE11	ARG123	1.821		ARG24	GLY130	1.901
	LEU8	GLN99	2.465		SER11	ALA181	2.034
	SER9	THR103	2.145		THR4	THR184	2.032
	ASP12	ALA121	1.935	csAVP9	ARG22	ASN111	2.118
	SER13	THR127	2.205		ALA11	GLU134	2.101
	ASN18	GLY183	2.724	csAVP10	SER4	THR178	2.350
csAVP5	ALA11	GLU134	2.101		SER4	GLN177	2.478
	ARG22	ASN111	2.118		ARG6	GLN177	2.094

**Table 6:** Calculated binding energy, docking scores, binding affinity (ΔG), and dissociation constant (Kd) from molecular docking of ten candidate peptides (csAVP1 to csAVP10) to SARS-CoV-2 3CLpro by PROGIDY and PIMA web server. Molecular docking scores, H-bond, electrostatic, and van der Waals energy (H-bond Ener., Elec. Ener., and VDW. Ener.) are presented in kJ/mol unit.

Peptide ID	Binding Energy (kJ/mol)			Docking Scores (kJ/mol)		Binding Affinity and Dissociation Constant	
	H-Bond. Ener.	Elec. Ener.	VDW. Ener.	GalaxyPepDock	HPEPDOCK	ΔG (kcal/mol)	Kd (M) at 25.0 °C
csAVP1	−31.64	4.46	−140.78	−167.96	−202.55	−11.0	9 × 10^−9^
csAVP2	−34.55	0.00	−114.22	−148.76	−162.16	−9.4	1.2 × 10^−7^
csAVP3	−24.70	8.04	−183.80	−200.46	−182.57	−13.9	6.10 × 10^−11^
csAVP4	−41.90	15.53	−420.90	−447.27	−203.25	−18.2	4.30 × 10^−14^
csAVP5	−12.54	0.00	−215.81	−228.36	−193.07	−13.4	1.60 × 10^−10^
csAVP6	−69.15	−6.44	−496.58	−572.17	−158.42	−15.6	3.40 × 10^−12^
csAVP7	−28.83	0.46	−94.16	−122.52	−167.37	−9.4	1.20 × 10^−7^
csAVP8	−39.60	−5.23	−286.11	−330.94	−212.76	−13.8	7.00 × 10^−11^
csAVP9	−12.16	−24.15	−180.11	−216.42	−229.77	−11.4	4.70 × 10^−9^
csAVP10	−10.42	2.17	−101.72	−109.97	−218.87	−8.2	9.50 × 10^−7^

## Data Availability

Not applicable.

## Supplementary Materials

The following supporting information can be downloaded at: https://www.mdpi.com/article/10.3390/molecules28010050/s1, Table S1: Total peptide sequences from the putative trypsinized peptidome of hemp seed (*Cannabis sativa*) and Table S2: Similarity analysis results by BLAST search against the peptides stored in anti-coronavirus peptides database (ACovPepDB). For transparency, all raw data from the re-analysis, including the input FASTA file and the AVP-IC_50_Pred server outputs (with job IDs), are available in a public GitHub repository: https://github.com/pramotec/supplementary-https-doi.org-10.3390-molecules28010050 (accessed on 30 September 2026).
