# Correction: Prasertsuk et al. Computer-Aided Screening for Potential Coronavirus 3-Chymotrypsin-like Protease (3CLpro) Inhibitory Peptides from Putative Hemp Seed Trypsinized Peptidome. *Molecules* 2023, *28*, 50

**DOI:** 10.3390/molecules31213959

**Published:** 2026-11-03

**Authors:** Kansate Prasertsuk, Kasidit Prongfa, Piyapach Suttiwanich, Nathaphat Harnkit, Mattanun Sangkhawasi, Pongsakorn Promta, Pramote Chumnanpuen

**Affiliations:** 1 Pibulwitthayalai School, 777 Naraimaharach, Talaychoopsorn, Lopburi District, Lopburi 15000, Thailand; 2 Medicinal Plant Research Institute, Department of Medical Sciences, Ministry of Public Health, Nonthaburi 11000, Thailand; 3 Program in Biotechnology, Faculty of Science, Chulalongkorn University, Bangkok 10330, Thailand; 4 Omics Center for Agriculture, Bioresources, Food and Health, Kasetsart University (OmiKU), Bangkok 10900, Thailand; 5 Department of Zoology, Faculty of Science, Kasetsart University, Bangkok 10900, Thailand

**Error in Table 2**

In the original publication [1], there was a mistake in Table 2 as published. Concerns regarding the accuracy of the data in Table 2 were brought to the attention of the Editorial Office, and the authors subsequently conducted a re-analysis of the data. As the original output files from the 2022 analysis were no longer accessible, the IC_50_ predictions for csAVP1–csAVP10 were re-run using the AVP-IC50Pred server, with the peptide sequences listed in Table 1 and the same settings as the original analysis (virus-specific prediction mode RSV/INFV/HSV; Hybrid Models I and II; default parameters); repeated runs gave identical values. The re-analysis showed that several values in the published Table 2 could not be reproduced. The virus-specific Random Forest (RF) values had been reported as 0.01 µM for all ten peptides, whereas the re-analysis gave values of 14.62–366.97 µM, and the Hybrid Model II IBk values did not correspond to the server output, which gave 0.01 µM for all ten peptides. Several other SVM, RF, and IBk values also differed from the re-analysis, most notably for csAVP1, while the K Star values were unchanged. Table 2 has therefore been replaced in full with the values obtained from the re-analysis, and the average IC_50_ values in the last column, calculated as the mean of the ten predicted values, have been recalculated accordingly. The corrected Table 2 appears below.

**Table 2:** Predictive AVP half maximal inhibitory concentration (IC_50_) by SVM, RF, IBk, and K Star-based models from the AVP-IC_50_Pred webserver.

Peptide ID	Virus Specific		Hybrid Model I *				Hybrid Model II *				Average IC_50_ (µM)
	SVM	RF	SVM	RF	IBk	K Star	SVM	RF	IBk	K Star	
csAVP1	46.17	30.19	99.44	38.00	125.46	0.22	99.26	38.54	0.01	0.01	47.73
csAVP2	45.82	75.32	78.69	108.68	152.62	11.41	78.60	109.07	0.01	0.01	66.02
csAVP3	45.90	111.35	32.97	41.74	58.84	17.01	33.05	42.34	0.01	0.01	38.32
csAVP4	45.92	104.83	40.75	19.24	19.85	8.01	40.74	19.62	0.01	0.01	29.90
csAVP5	48.00	14.62	49.27	19.61	33.46	11.01	49.19	19.84	0.01	0.01	24.50
csAVP6	45.92	247.86	39.43	73.22	35.16	0.01	39.45	71.64	0.01	0.01	55.27
csAVP7	45.79	126.57	46.57	49.41	19.85	313.01	46.56	49.17	0.01	0.01	69.70
csAVP8	45.91	171.33	38.71	13.64	11.80	1.01	38.74	13.84	0.01	0.01	33.50
csAVP9	45.75	366.97	45.99	56.50	36.75	14.01	45.91	56.50	0.01	0.01	66.84
csAVP10	45.92	185.64	45.75	17.61	62.98	9.01	45.73	17.63	0.01	0.01	43.03

Note: Hybrid Model I * = Composition(mono-di) + Physico + Secondary structure + Surface accessibility; Hybrid Model II * = Binary(N8/C8) + Physico + Secondary structure + Surface accessibility.

**Text Correction**

There was an error in the original publication. Because the predicted IC_50_ values in Table 2 have been corrected, the statements based on these values have also been corrected. A correction has been made to 2. Results and Discussion, 2.2. Predictive Antiviral Scores, IC_50_, and Physicochemical Properties of the Selected csAVPs, Paragraph 3:

From the virus-specific SVM model prediction, all csAVPs were found to have quite comparable predicted IC_50_ (45–48 µM), whereas the virus-specific RF model gave a wider range of predicted IC_50_ (14.62–366.97 µM). The higher predicted IC_50_ of csAVP1 and csAVP5 above 46 µM in the virus-specific SVM model indicated lower antiviral efficacy compared to other peptide candidates. However, csAVP5 and 4 were found to have the highest efficacy among putative antivirals based on the average IC_50_ across three prediction models, with only 24.50 and 29.90 µM, respectively.

A correction has been made to 2. Results and Discussion, 2.2. Predictive Antiviral Scores, IC_50_, and Physicochemical Properties of the Selected csAVPs, Paragraph 4:

However, all csAVPs could be classified as highly effective (<1 µM) if only the Hybrid Model II IBk and K Star models were considered, as could csAVP1 and csAVP6 based on the Hybrid Model I K Star model. Based on the virus-specific SVM model, the predicted antiviral efficacy of our selected csAVPs is comparable to that of the selected fruit bromelain-derived peptide for human angiotensin-converting enzyme 2 (ACE-2) inhibition (40.67 µM), whereas the virus-specific RF values of our csAVPs (14.62–366.97 µM) are higher than that of the bromelain-derived peptide (6.85 µM) [30]. 

A correction has been made to 4. Conclusions, Paragraph 1:

csAVP4 was selected as the best candidate for coronavirus 3CLpro inhibition among all selected csAVPs based on its predicted efficacy (the second-lowest average predicted IC_50_ among the selected csAVPs), high affinity and binding energy, and low possible side effects to the host cell.

**Supplementary Materials**

For transparency, all raw data from the re-analysis, including the input FASTA file and the AVP-IC_50_Pred server outputs (with job IDs), are available in a public GitHub repository: https://github.com/pramotec/supplementary-https-doi.org-10.3390-molecules28010050 (accessed on 30 September 2026).

The authors state that the scientific conclusions are unaffected. This correction was approved by the Academic Editor. The original publication has also been updated.
