# Supplementary material for: Computer-Aided Screening for Potential Coronavirus 3-Chymotrypsin-like Protease (3CLpro) Inhibitory Peptides from Putative Hemp Seed Trypsinized Peptidome
Source: Molecules. 2022 Dec 21;28(1):50. doi: 10.3390/molecules28010050 (PMC9822321; doi:10.3390/molecules28010050)
Supplement: Supplementary file 1 [file molecules-28-00050-s001.zip › molecules-2033820-supplementary.pdf]

Table S1. Total peptide sequences from the putative trypsinized peptidome of hemp seed (*Cannabis sativa*).

ID	Sequences	length (aa)	original protein
seq1	EQGQDK	6	Albumin
seq2	MMESAR	6	Albumin
seq3	QDLNHCR	7	Albumin
seq4	TCQFHSR	7	Albumin
seq5	SGSEQECR	8	Albumin
seq6	TTITTECR	8	Albumin
seq7	CPALEMEIQK	10	Albumin
seq8	NIPSMCGMQPR	11	Albumin
seq9	TTITTVETDDVENYSR	16	Albumin
seq10	FEEEDEIENYSQHLDQCCSQLR	22	Albumin
seq11	ISSSTLALFAALMLVAHAVAFR	22	Albumin
seq12	EIEQER	6	Edestin1
seq13	CFDGEVR	7	Edestin1
seq14	GTLDLVR	7	Edestin1
seq15	HSSENYR	7	Edestin1
seq16	SQDESYR	7	Edestin1
seq17	SSQEHQR	7	Edestin1
seq18	ENIGDPSR	8	Edestin1
seq19	FLQLSAER	8	Edestin1
seq20	LQGQNDDR	8	Edestin1
seq21	QQNQCQIDR	9	Edestin1
seq22	ADVFTPQAGR	10	Edestin1
seq23	GTLDLVSPLR	10	Edestin1
seq24	VQVVNHMGQK	10	Edestin1
seq25	EVTVPQNHAVVK	12	Edestin1
seq26	EETVLLTSSTSSR	13	Edestin1
seq27	ISTVNSYNLPILR	13	Edestin1
seq28	QASSDGFEWVSFK	13	Edestin1
seq29	TNDNAWVSPLAGR	13	Edestin1
seq30	GQGQGQSQGSQPDR	14	Edestin1
seq31	YEANGLEETFCSMR	14	Edestin1
seq32	YLEEAFNVDSETVK	14	Edestin1
seq33	YPEEAFNVDSETVK	14	Edestin1
seq34	ALPEAVLANAFQISR	15	Edestin1
seq35	FYLAGNPEDEFEQLR	15	Edestin1
seq36	QGQIVTVPQNHAVVK	15	Edestin1
seq37	GQGQGQSQGSQPDGGR	16	Edestin1
seq38	QGQINQFQCAGVAVVR	16	Edestin1
seq39	GILGVTFPGCPETFEESQR	19	Edestin1
seq40	VEAEAGLIESWNPNHSPLR	19	Edestin1
seq41	NAIYTPHWNVNAHSVMYVLR	20	Edestin1
seq42	NAIYTPHWNVSAHSVMYVLR	20	Edestin1
seq43	ALLSLSFCFFLLLQGTSAISR	21	Edestin1
seq44	YTIEQNGLHLPSYTNTPQLVYIVK	24	Edestin1
seq45	YTIQQNGLHLPSYTNTPQLVYIVK	24	Edestin1
seq46	VEAEAGLIESWNPNHSQFQCAGVAVVR	27	Edestin1

ID	Sequences	length (aa)	original protein
seq47	EGDIVAIPAGVAYWSYNNGDQQLVFVSLLDTSNVNNQLDDNPR	43	Edestin1
seq48	IQEEER	6	Edestin2
seq49	NPLAGK	6	Edestin2
seq50	TQQQVR	6	Edestin2
seq51	TNDNAMR	7	Edestin2
seq52	FLQLTAER	8	Edestin2
seq53	GTSSPSSR	8	Edestin2
seq54	NVFDGELR	8	Edestin2
seq55	FHLAGNPHR	9	Edestin2
seq56	LQVVDDNGR	9	Edestin2
seq57	WQSQCQFQR	9	Edestin2
seq58	PSQADIFNPR	10	Edestin2
seq59	GEDLQIIAPSR	11	Edestin2
seq60	ASAQGFEWIAVK	12	Edestin2
seq61	DNGLEETFCTLR	12	Edestin2
seq62	SEGASSDEQHQK	12	Edestin2
seq63	LNTLNNYNLPILR	13	Edestin2
seq64	DEISVFSPSSQQTR	14	Edestin2
seq65	AMPDDVLANAFQISR	15	Edestin2
seq66	EGQIFVVPQNFAVVK	15	Edestin2
seq67	ILAESFNVDTELAHK	15	Edestin2
seq68	GIHGAVIPGCPETFER	16	Edestin2
seq69	ESGEQTPNGNIFSGFDTR	18	Edestin2
seq70	GLLLPSFLNAPMMFYVIQGR	20	Edestin2
seq71	NGMMAPHFNLDSHSVIYVTR	20	Edestin2
seq72	SSTSLLCFTLFSLLLSHACFAQIEQMPQR	29	Edestin2
seq73	VECEAGVSEYWDIQNTEDDELHCAGVETAR	30	Edestin2
seq74	EGDMVAMPAGVADWVYNNGDSPLVLIAFVDVGNQANQLDQFSR	43	Edestin2
seq75	ESMGDPAR	8	Edestin3
seq76	IQNQDDFR	8	Edestin3
seq77	QELQQTER	8	Edestin3
seq78	QEQEQEMR	8	Edestin3
seq79	SVFEGEIR	8	Edestin3
seq80	AQVNQLAGK	9	Edestin3
seq81	CQVVDNNGR	9	Edestin3
seq82	EGFEWVSFK	9	Edestin3
seq83	LEACEPDHR	9	Edestin3
seq84	ADVFSPQAGR	10	Edestin3
seq85	EQEGLPNNVFR	11	Edestin3
seq86	DYNGLEENICTMR	13	Edestin3
seq87	EESSLFTTSHQGIR	14	Edestin3
seq88	TAMYGDQNECQLNR	14	Edestin3
seq89	AMPEDVIANSYQISR	15	Edestin3
seq90	QGQALTVPQNFAVVK	15	Edestin3
seq91	LTTVNSYNLPILSFLR	16	Edestin3
seq92	GFSVNLIQEAFNVDSETAR	19	Edestin3
seq93	NAMYAPQYTMNAHNIIYAIR	20	Edestin3
seq94	FYIAGNPHQEFPQSMMTQQGR	21	Edestin3
seq95	LTIQPNGLHLPSYTNGPQLIHVIR	24	Edestin3

ID	Sequences	length (aa)	original protein
seq96	MASTPLLLSLSLCFLVLLHGCSAR	24	Edestin3
seq97	GVLGTLFPGCAETFEEAQVSVGGGR	25	Edestin3
seq98	VECEGGMIESWNPNHEQFQCAGVALLR	27	Edestin3
seq99	EGDIIAIPAGMAYWCNNDGDQPLITVNLIHIINNQNQLDMSPR	43	Edestin3
seq100	DQLAER	6	Vicilin
seq101	LGFIYK	6	Vicilin
seq102	TGDVYR	6	Vicilin
seq103	TTWSWR	6	Vicilin
seq104	FAQGPLR	7	Vicilin
seq105	TDAGEMR	7	Vicilin
seq106	GELPFILR	8	Vicilin
seq107	ATEYGIILK	9	Vicilin
seq108	EGDVFWVPR	9	Vicilin
seq109	IGFITMEPK	9	Vicilin
seq110	LGNLTSYQR	9	Vicilin
seq111	LLDSLFANR	9	Vicilin
seq112	YFPFCQIASR	10	Vicilin
seq113	SPDSYNLYDGK	11	Vicilin
seq114	PQFLVGASSILR	12	Vicilin
seq115	AGPMEFFGFTTSAR	14	Vicilin
seq116	GPELAAAFGLSLER	14	Vicilin
seq117	LHIICSFDTSESLR	14	Vicilin
seq118	ESVILPTSAASPPVK	15	Vicilin
seq119	ISAGSAFYLVNTGEGQR	17	Vicilin
seq120	TLFLPQYLDSELTIFIR	17	Vicilin
seq121	EILSSQQEGPIVYIPDSR	18	Vicilin
seq122	NNYGWSIALDEFSYSPLR	18	Vicilin
seq123	GTGMIQVVYPNGTSAMNTEVK	21	Vicilin
seq124	SGCGVYLVNLTAGSMMAPHLNPK	23	Vicilin
seq125	IVGFHQGEEEEDEEELEEDINQEQNQK	27	Vicilin
seq126	LTLLVLMLVLSYGVLGIMGFDEDEDWTR	28	Vicilin
seq127	IGTFQSFFLGGGTNPASILSGFDSEILENAFNVTHAELK	39	Vicilin

Table S2. Similarity analysis results by BLAST search against the peptides stored in anti-coronavirus peptides database **(**ACovPepDB**)**

Query Peptide (csAVP4)	Similar Peptide in ACovPepDB		%Matching	%Identity
	ID	Sequences		
IGTFQSFFLGGGTNPASILSGFDSEILENAFNVTHAELK	AcoVP100440	DKEWILQKIYEIMRLLDELGHAEASMRVSDLIYEFMKKGDERLLEE AERLLEEVER	30.77	26
IGTFQSFFLGGGTNPASILSGFDSEILENAFNVTHAELK	AcoVP100262	SLTHRKFGGSGGSPFSGLSSIAVRSGSYLDAIIDGVHHGSGGNLSPTF TFGSGEYISNMTIRSGDYIDNISFETNMGRRFGPYGGSGGSANTLSN VKVIQINGSAGDYLDSLDIYYEQY	28.21	45
IGTFQSFFLGGGTNPASILSGFDSEILENAFNVTHAELK	AcoVP100196	PTTFMLKYDENGTITDAVDC	28.21	36
IGTFQSFFLGGGTNPASILSGFDSEILENAFNVTHAELK	AcoVP100195	FKLPLGINITNFRAILTAFS	25.64	50
IGTFQSFFLGGGTNPASILSGFDSEILENAFNVTHAELK	AcoVP100151	EEQAKTFLDKFNHEAEDLFYQSSGLGKGDFR	23.08	56
IGTFQSFFLGGGTNPASILSGFDSEILENAFNVTHAELK	AcoVP100223	MWKTPTLKYFGGFNFSQIL	23.08	56
IGTFQSFFLGGGTNPASILSGFDSEILENAFNVTHAELK	AcoVP100199	RDVSDFTDSVRDPKTSEILD	12.82	80
IGTFQSFFLGGGTNPASILSGFDSEILENAFNVTHAELK	AcoVP100511	TDAVDCSQNPLAELKCSVKSF	10.26	100
